# Coral individuality – confluence of change physical splitting and developmental ability of embryos

**DOI:** 10.1038/s41598-017-16273-w

**Published:** 2017-11-22

**Authors:** Nami Okubo, Sho Toshino, Yoshikatsu Nakano, Hiromi H. Yamamoto

**Affiliations:** 1 Department of Economics, Tokyo Keizai University, 1-7-34 Minamimachi, Kokubunji, Tokyo, 185-8502 Japan; 2 Tropical Biosphere Research Center, Sesoko Station, University of the Ryukyus, 3422 Sesoko, Motobu, Okinawa, 905-0227 Japan; 3 Ocean Expo Research Center, Motobu, Okinawa, Japan

## Abstract

Previous studies have suggested that blastomeres from the 2-, 4-, or 8-cell stage of corals have the ability to develop into normal primary polyps. However, it is still not known which developmental stage’s blastomere produces which juvenile. In this study, we demonstrated that only the blastomeres with animal hemispheres have the capacity to develop into normal primary polyps. Individuality was evaluated using blastomeres isolated from the corals *Acropora digitifera*, *A. intermedia*, *Dipsastraea lizardensis*, and *Favites chinensis*. On commencement of embryo cleavage, the animal pole was marked using Neutral red staining, and at the 2-, 4-, and 8-cell stages, embryos were divided into individual blastomeres using glass needles. We found that the survival rate and percentage metamorphosis were higher in the larger-sized blastomeres with animal hemispheres. The vegetal hemisphere alone is incapable of developing into a normal primary polyp; however, a ball-shaped embryo with incomplete mesenteries and no pharynx developed in some cases. These results indicate that the animal hemisphere is needed for corals to develop into normal primary polyps, and that the individuality of corals is possibly determined by a combination of the chance physical splitting of embryos by waves and their innate developmental ability.

## Introduction

The phylum Cnidaria comprises the five classes Anthozoa, Cubozoa, Hydrozoa, Scyphozoa, and Staurozoa. Anthozoan species, which include the Hexacorallia (hard corals and sea anemones) and Octocorallia (soft corals, sea pens, and gorgonians)^1^, have no alternation of medusae in their life cycles, whereas species in the classes Cubozoa (box jellies or sea wasps), Hydrozoa (hydras, hydroids, hydromedusae, and siphonophores), Scyphozoa (true jellyfish), and Staurozoa (stalked jellyfish), which are classified in the subphylum Medusozoa, show alternation of generations between an asexually reproducing benthic polyp and a sexually reproducing planktonic medusa^2–5^.

Because the phylum Cnidaria is the evolutionary sister group of Bilateria and plays an important role in our understanding of the evolutionary history between Diploblasts and Triploblasts, the study of model animals within each class of Cnidaria, such as *Podocoryne carnea* and *Nematostella vectensis*, is well developed. Although cnidarians show variability in cleavage patterns and modes of gastrulation, they have the following characteristics in common: the eggs have a clear animal-vegetal polarity and the animal pole becomes the oral part^6^.

Compared with these cnidarian model animals, information on hard corals in the order Scleractinia are currently limited, primarily because rearing corals in closed aquaria is difficult. Moreover, the spawning of corals occurs only once or twice a year and the timing of these events is difficult to predict. However, there is an increasing need to gain further information on the basic developmental biology of various coral species, particularly sexual propagation, to support conservation measure for coral reefs, which are under mounting pressure from artificial and/or natural disturbances.

To this end, Okubo *et al*.^7–9^ recently described the embryogenesis of various types of corals. During the development of different coral species, particularly from the 2- to the 64-cell stage, embryos are often observed to split into a few blastomeres, which become tiny planulae and juveniles. This phenomenon, termed “polyembryony,” is the physical splitting of cells after the first few mitotic divisions into separate embryos^10^. Polyembryony occurs during embryogenesis after gametogenesis, and therefore differs from the parental manipulation of offspring size to maximize either their own fitness or that of the offspring^11^. Animals exhibiting polyembryony include Cnidarians, Platyhelminthes, Arthropoda, Bryozoa, Echinodermata, and Chordata^10^, and although its evolutionary significance has been discussed, this remains unclear because polyembryony does not appear to be necessarily advantageous for either sexual or asexual reproduction.

Within the phylum Cnidaria, natural polyembryony has been observed in settled hydroid colonies: *Pegantha* spp.^12^, *Cunina* spp.^12,13^, and *Cunoctantha* spp.^12^ in the order Narcomedusae and *Polypodium hydriforme* ^14,15^ in the order Polypodiidae. However, no information is available on hydrozoan embryo polyembryony in nature. In Anthozoa, two studies have reported polyembryony of corals in nature based on analysis using microsatellite markers^16,17^. Under experimental conditions, Heyward and Negri^18^ reported that fragmented embryos, i.e., blastomeres from the 2-, 4-, or 8-cell stage, have the ability to become normal juvenile polyps in corals; however, it is still not known which stage’s blastomere produces which juvenile.

Momose and Schmid^19^ revealed that in the hydrozoan jellyfish *Podocoryne carnea*, the organizer that determines oral–aboral axis polarity and endodermal cell fate is localized in the animal pole. Within the class Anthozoa, molecular marker analysis, using *NvFoxA, NvWnt2*, and *NvFGF1A*, has indicated that in the sea anemone *Nematostella vectensis*, an organizer localized within the animal pole promotes normal polyp development, and that the correct patterning of the aboral pole depends on signals from the oral half^6^. Morphologically, the third cleavage into the 8-cell stage in corals occurs equatorially, and thereafter the four lower cells are formed from the vegetal hemisphere, and the pharynx is formed at the animal pole^7,9,20^. On the basis of the results of these previous molecular and morphological studies in *P. carynera* and *N. vectensis*, it could be hypothesized that in corals only the blastomeres with an animal hemisphere can develop into normal primary polyps.

**Figure 1:**
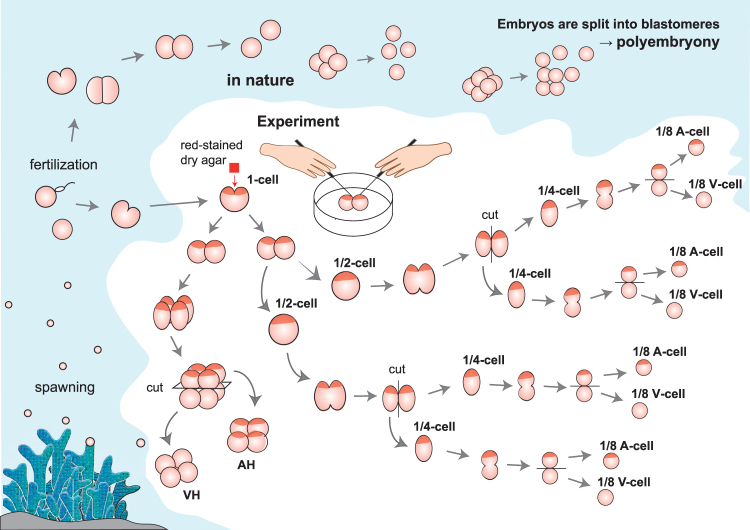
Diagram of polyembryony and the experimental procedure used in the present study. A: embryos with an animal hemisphere but without a vegetal hemisphere, V: embryos with a vegetal hemisphere but without an animal hemisphere.

In the present study, we investigated the fate of individual coral blastomeres that had been artificially divided (Fig. 1), and demonstrated that only the blastomeres with animal hemispheres have the capacity to develop into normal primary polyps.

## Results

**Figure 2:**
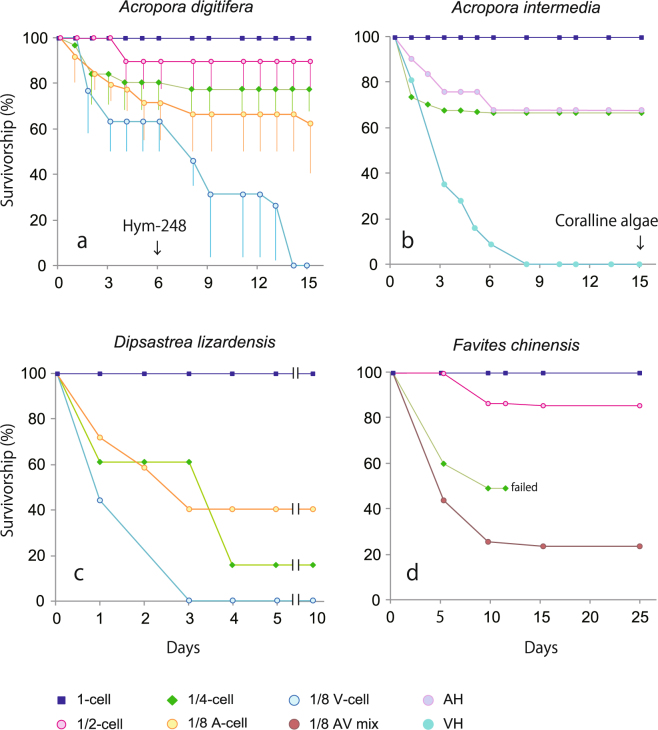
Survival rate of the control embryos and blastomeres divided in the 2–8-cell stages. (**a**) *Acropora digitifera*, (**b**) *Acropora intermedia*, (**c**) *Dipsastraea lizardensis*, (**d**) *Favites chinensis*. A: embryos with an animal hemisphere but without a vegetal hemisphere, V: embryos with a vegetal hemisphere but without an animal hemisphere, AH: animal-half hemisphere (1/2 embryo), VH: vegetal-half hemisphere (1/2 embryo). Bar: S.D.

A decrease in the survival rate of embryos was apparent during the first 4 days after splitting of embryos (Fig. 2); however, it rarely changed during the rest of the observation period. The embryos derived from vegetal hemispheres had a lower survival rate than those derived from animal hemispheres (Fig. 2a,b,c, P < 0.01 in *A.digitifera*). In *D. lizardensis*, all the embryos derived from the vegetal hemisphere died within 9 days, whereas 62% of embryos derived from the animal hemisphere survived (Fig. 2c). Similarly, in *A. digitifera*, the blastomeres with an animal hemisphere from the 8-cell stage had a higher survival rate than those with a vegetal hemisphere. Furthermore, the larger-sized blastomeres had a higher survival rate than the smaller blastomeres (Fig. 2b,c,d). In *A. digitifera*, however, no significant difference was observed among survival curves of 1/2, 1/4 and 1/8-cells from animal hemisphere, although the survival curve of 1-cell was significantly higher than those of the smaller cells (Bonferroni multiple comparison test, P < 0.01). The animal- and vegetal-half embryos (Fig. 2b) were the same size as 1/2-cells; however, the survival rate of the animal-half embryos was similar to that of 1/4-cells, whereas that of the vegetal-half embryos was considerably lower.

We used Hym-248 (a member of the LW amide family)^21^ and coralline algae^22^ for induction of metamorphosis in *A. digitifera*. The neuropeptide Hym-248 acts hormonally to induce metamorphosis in some hydrozoan species and in corals of the genus *Acropora* ^21^. Hym-248 induced metamorphosis only in the control and 1/2-cells (Fig. 1). However, immediately after the application of this peptide at day 6 after fertilization, the survival rate of 1/8-cells from vegetal hemisphere decreased sharply (Fig. 2, Table 1). In contrast, in *A. intermedia*, coralline algae induced metamorphosis in the blastomeres with an animal hemisphere (Table 1). Hence, the sharp decrease in survival rate *A. digitifera* embryos could be attributable to the lower tolerance to chemicals in the smaller embryos. In the other two species, metamorphosis occurred without artificial induction. In *D. lizardensis*, the control and 1/2-cells metamorphosed, whereas in *F. chinensis*, the blastomeres with an animal hemisphere, including 1/8-cells, metamorphosed (Fig. 1, Table 1).

**Table 1:** Percentage survival and metamorphosis after the cell division experiment.

	*Acropora. digitifera* (15 d)			*Acropora intermedia* (25 d)			*Dipsastraea lizardensis* (17 d)			*Favites chinensis* (11 d)		
	N	S	M	N	S	M	N	S	M	N	S	M
		%	Hym		%	alg		%	—		%	—
1 (Control)	30	**100**	**100**	21	**90**	**68**	30	**100**	**37**	30	**100**	**10**
1/2	22	**82** ± **11**	**58** ± **30**	—	—	—	30	**100**	**53**	60	**97**	**2**
1/4	49	**68** ± **7**	**0**	67	**84**	**52**	30	**30**	**0**	76	**56**	**4**
1/8 A	24	**63** ± **12**	**0**	19	**47**	**22**	30	**13**	**0**	—	—	—
1/8 V	19	**0**	**0**	8	**13**	**0**	30	**10**	**0**	—	—	—
1/8 AV mix	—	—	—	26	**42**	**27**	—	—	—	35	**29**	**3**
AH	—	—	—	12	**67**	**50**	—	—	—		—	—
VH	—	—	—	12	**8**	**0**	—	—	—		—	—

N: sample number at the beginning of the experiment, S: survivorship, M: percentage metamorphosis, Hym: Hym 248 treatment, alg: coralline algae were placed in the culture dish, AH: animal hemisphere, VH: vegetal hemisphere. Percentage survival and metamorphosis were calculated as: number/N.

**Figure 3:**
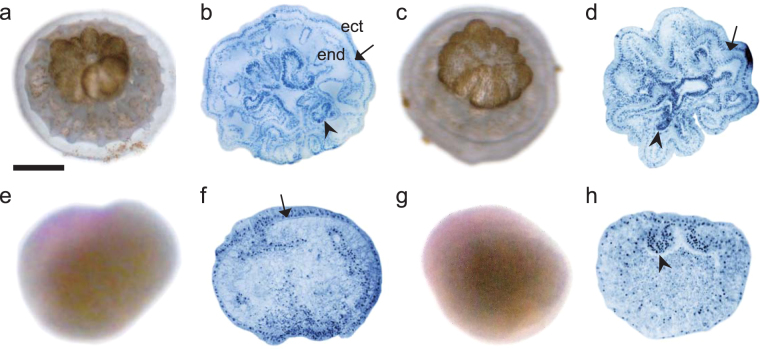
*Acropora digitifera*. (**a**) Polyp metamorphosed from a control embryo. (**b**) Section of Fig. 3a. (**c**) Polyp metamorphosed from a blastomere divided from a 2-cell stage. (**d**) Section of Fig. 1b. (**e**) Section of the planula, which did not metamorphose, from a blastomere divided from the 4-cell stage after the addition of Hym-248. (**f**) Ball-shaped planula derived from an animal hemisphere blastomere divided from the 8-cell stage after the addition of Hym-248. (**g**) Section of an animal hemisphere planula. (**h**) Section of a vegetal hemisphere planula. Scale bar represents 200 µm for (**a**,**b**); 100 µm for (**c**,**d**); 50 µm for (**e**); and 25 µm for (**f**–**h)**. The red part of the embryo in (**f**) indicates animal hemisphere staining. ect: ectoderm, end: endoderm, arrows: mesoglea, arrow heads: mesenteries.

Normally, gastrulation (two-germ layer formation) occurs until the third day post-fertilization, when embryos develop into planulae. During the planula stage, a pharynx should be formed in both modes of embryogenesis, and thereafter the planulae settle^7,23^. In our experiments, the controls and blastomeres from the 2-cell stage developed into elongate planulae, and some of these metamorphosed and settled (Table 1, Fig. 3a–e). However, in *A. intermedia*, 35% of the blastomeres from the 4-cell stage did not elongate and the embryos were ball-like or abnormally shaped with an irregular surface. Similarly, the blastomeres from the 8-cell stage that did not metamorphose or settle did not elongate and became ball shaped (Fig. 3f). Histological sections showed that no pharynx formed within the abnormally shaped embryos; however, endodermal- and ectodermal-like cells could be distinguished by mesoglea in the blastomeres containing an animal hemisphere from the 4- and 8-cell stages (Fig. 3e,g), whereas no mesoglea was formed in those derived from the vegetal hemisphere (Fig. 3h). These abnormal embryos continued swimming without definite direction or remained motionless until death (Fig. 3g).

## Discussion

Although it is not yet known which stage’s blastomere produced which juvenile in corals, our experiments indicated that the blastomeres containing an animal hemisphere can develop into a primary polyp (Fig. 2, Table 1). In the present study, only one blastomere with an animal hemisphere from the 8-cell stage of *A. intermedia* survived and metamorphosed (Table 1); therefore, in nature, few blastomeres derived from 1/8-cells would be expected to survive.

For corals and related animals, there is currently no information available on the cause of the higher mortality in embryos derived from vegetal hemispheres compared with those derived from animal hemispheres. The size of animal-half and vegetal-half embryos is the same as 1/2-cells (Fig. 1); however, the survival rate of those derived from the vegetal hemisphere was lower (Fig. 2b). To the best of our knowledge, there is no information available on the comparative survival rates of blastomeres with an animal or vegetal hemisphere. Since the survival of all the species examined in the present study apparently decreased in the first 4 days after the experiment (Fig. 2), during which time gastrulation occurred and oxygen consumption peaked^23,24^, it is conceivable that abnormal development disturbs metabolic activity.

Similar to our study on species in the class Anthozoa, observations on *N. vectensis* by Fritzenwanker *et al*.^6^ indicated that the vegetal halves could not develop into normal polyps. Although the embryos in this latter study had no external marker of the animal pole, the authors based their assumption on the fact that one-third of blastomeres should contain an animal and vegetal hemisphere, respectively, if each of the three division planes at the 8-cell stage is randomly cut^6^. In this case, the transcripts of the oral markers *NvFoxA* and *Novant* were not expressed in embryos derived from the vegetal hemisphere^6^. It is therefore possible that in corals, the genes for endoderm, axis, and pharynx formation cannot function without the animal hemisphere.

Among the species examined in the present study, the percentage metamorphosis in the control of *A. intermedia* was 68% lower than that in *A. digitifera* (Table 1). The manual of the Akajima Marine Science Institute (http://www.amsl.or.jp/etc/english.pdf), which was the first institute to successfully mass culture *Acropora* corals, states that acroporid larvae take 5–5.5 days to metamorphose and settle at 26 °C–27 °C. For example, *A. tenuis* has competency to settle for >20 days, whereas the competence decreases after 2 weeks in *A. nasuta*. Therefore, in the present study, there is a strong possibility that the time of metamorphosis induction was too late at day 15 after fertilization in *A. intermedia*.

Within the embryos derived from the animal hemisphere, no relationship was observed among blastomere sizes during metamorphosis (Table 1). However, Marshall and Bolton^25^ described that in two ascidian species and one sea urchin species, small increases in egg size dramatically increased developmental time until hatching. In the present study, the planulae that were not able to settle in the late experimental period gradually died, and the smaller embryos had a lower survival rate than the larger embryos (Fig. 2). This could be because the amounts of body constituents, such as lipids, were lower in the smaller blastomeres. Many studies have shown the effect of offspring size in marine invertebrates, including corals, on longevity and the competency period for settlement. For instance, Isomura and Nishihira^26^ found that larger planulae in pocilloporiid corals had longer lifetimes, whereas Marshall and Keough^27^ described that larger larvae delayed settlement for longer without settlement cues, and thus larger larvae could have greater dispersal potential than smaller larvae.

Considering the findings of these previous studies and our results showing the lower survival and genetic diversity of smaller embryos, it could be assumed that polyembryony is disadvantageous. A further factor that should perhaps be taken into consideration in relation to polyembryony is seawater salinity. In Micronesia and Okinawa, coral spawning takes place during the rainy season and although the salinity of coral reef seawater is normally approximately 35‰, under conditions of heavy rainfall, the discharge from streams and storm drains may reduce surface salinities over reefs to 25‰ to 26‰ or lower^28^. In this regard, it is known that low salinity induces polyembryony in the embryos of the sand dollar *Echinarachnius parma* and the pencil urchin *Eucidaris tribuloides* ^1^. In corals, low salinity has been observed to cause a 51% decrease in the number of embryos developing to the planulae stage^29^, and thus polyembryony could lead to a considerably lower survival rate of offspring. Furthermore, with the exception of *Tubastraea* ^8^, coral embryos have no fertilization membrane, and therefore the embryos of corals may split more readily than those of other marine invertebrates.

The foregoing assessment thus leads us to ask what is the advantage of polyembryony in corals? One possible explanation relates to sperm limitation in the marine environment. The number of sperm is diluted rapidly when corals release gametes into the water column, such that there is a low probability of egg and sperm meeting to facilitate fertilizations^30^. In such cases, if fertilized eggs are limited and thus split and divide into many, polyembryony could contribute to an increase in offspring number^10^, albeit clones. If sperm limitation is disadvantageous to corals, species with self-fertilization might not have polyembryony because they are fertilized internally and the sperm was not diluted by seawater. However, the findings of the two previous studies on *Pocillopora damicornis* described above do not concur with this possibility. In *P. damicornis*, which is an internal fertilizer, one pair of sexually produced, genetically identical larvae were found in Taiwan^16^ and sexually produced, genetically identical siblings (two pairs of twins and one triplet) were found in Moorea, French Polynesia^17^, indicating the possible occurrence of polyembryony.

The other possible advantage of polyembryony is that larvae of one genotype could disperse widely and settle in various micro-scale niches depending on blastomere size. For example, from the 4-cell stage, the embryo could possibly be broken unevenly, i.e., a 4-cell stage could be broken into one blastomere and three attached blastomeres. Therefore, one small larva and one large larva of a single genotype would be produced. Coral larvae prefer to settle in micro-crevices^31^, and thus smaller larvae could settle in smaller crevices, more efficiently thereby avoiding attacks from predators.

Consequently, for corals that disperse widely, it is still unclear whether polyembryony could have evolutionary significance. Normal sexual reproduction produces a variety of genotypes, which can facilitate adaptation to different environmental conditions, whereas polyembryony propagates only one unproven genotype (Craig *et al*. 1997). However, if the local environment does not change rapidly, asexual reproduction, such as polyembryony, may be more advantageous than sexual reproduction. Therefore, it would be interesting to study how environmental stresses, such as adverse salinity and pH, affect cell adjunction, which may be a cause of polyembryony. The information obtained in the present this study would be useful for the farming of sexually propagated corals and outplanting corals for reef rehabilitation. It would be better to avoid strong stirring of the water during early embryogenesis period. Otherwise, embryos are easily broken apart and survival rate of seedling would become lower as mentioned in this study.

In conclusion, the individuality of corals is possibly determined by a combination of the chance physical splitting of coral embryos by waves and their innate developmental ability.

## Materials and Methods

Experiments were conducted using *Acropora digitifera* and *A. intermedia* (from a complex clade) during the spawning season in 2009 and 2012 and in 2012, respectively, and *Dipsastraea lizardensis* and *Favites chinensis* (from a robust clade) during the spawning season in 2009 and in 2016, respectively^32^. We selected species from these two clades because the mode of development differs between the clades^7–9^. The *A. intermedia* used in the experiments were cultured in the Okinawa Churaumi Aquarium, Okinawa, Japan. This cultured coral has normal characterization and natural colonies^24^. The other experimental species were taken from natural colonies in Sesoko Island, Okinawa, Japan. After spawning, egg–sperm bundles from three different colonies of each species were inseminated together. When the first cleavage had occurred in *A. digitifera*, *A. intermedia*, and *D. lizardensis*, the animal pole was stained with Neutral red-stained dry agar to distinguish the locality of the animal and vegetal hemispheres in embryos from the 8-cell stage (Fig. 1). Neutral Red is a weak cationic dye that readily penetrates the cell membrane and accumulates intracellularly in lysosomes. The agar base was used to hold the embryo in place. The embryo was allowed to absorb the stain for approximately 30s, and then the agar was removed by washing the embryo with artificial seawater. The embryos were divided into blastomeres using glass micro-needles (tips of injection capillaries) at the 2-, 4-, and 8-cell stages (Fig. 1). These methods differ from those used by Heyward and Negri^18^, who gently poured embryos twice from one 2-L plastic container into another over a vertical distance of 30 cm, and thus they could not distinguish the polarity of each blastomere.

Blastomeres were maintained individually in single wells of a 24-well plate filled with 0.22-µm-filtered natural seawater (GSWP04700 MF Millipore), and survival rate and percentage metamorphosis were monitored once daily for 2 weeks under a microscope, and thereafter, a few times weekly for 1 month. Experimental replicates obtained at different spawning times could be obtained only for *A. digitifera*, although for all species gametes from three colonies were mixed for one experiment to avoid incompatibility of fertilization among colonies. Significant differences were analyzed using the Kaplan-Maier method. When a statistically significant difference was detected among the survival curves (P < 0.01), all pairwise multiple comparisons were performed using a log-rank test with the statistical significance level adjusted using by the Bonferroni method to determine differences between survival curves.

Since larval settlement and competency is not uniform among species, a metamorphosis inducer was applied when individuals had reached the elongate planula stage. For *A. digitifera*, beginning from day 6 after splitting of embryos, the *Hydra* peptide Hym-248 was added to each well to promote metamorphosis. However, in the first experiment using *A. digitifera*, all the 1/8 embryos (Fig. 1) died immediately after the treatment with Hym-248 (Fig. 2). Given the apparent detrimental effects of this hormone, we provided coralline algae in subsequent studies using *A. intermedia* at d 15 of the experiment. Although no treatment was conducted for settlement of the other species, they settled naturally on plastic plates.

On metamorphosis, the experimental blastomeres (either unsettled, or settled) were fixed in 10% formalin/90% filtered seawater for 2 h at room temperature (25–26 °C), to determine tissue development. Fixed samples were infiltrated with a graded series of ethanol (70–100%). The samples were embedded in glycol methacrylate (Technovit 7100; Heraeus Kulzer GmbH, Germany) and sectioned at a thickness of 10 µm using a microtome (Leica RM2125; Leica Microsystems). All sections were mounted on glass slides coated with γ-aminopropyltriethoxysilane and stained with methylene blue.

### Ethical approval

All applicable international, national, and/or institutional guidelines for the care and use of animals were followed.
